# Low seroprevalence of Zika virus infection among adults in Southern Taiwan

**DOI:** 10.1186/s12879-019-4491-4

**Published:** 2019-10-23

**Authors:** Yu-Wen Chien, Tzu-Chuan Ho, Pei-Wen Huang, Nai-Ying Ko, Wen-Chien Ko, Guey Chuen Perng

**Affiliations:** 1 Department of Public Health, College of Medicine, National Cheng Kung University, Tainan, Taiwan; 2 Department of Occupational and Environmental Medicine, National Cheng Kung University Hospital, College of Medicine, National Cheng Kung University, Tainan, Taiwan; 3 Institute of Basic Medical Sciences, College of Medicine, National Cheng Kung University, Tainan, Taiwan; 4 Department of Nursing, College of Medicine, National Cheng Kung University, Tainan, Taiwan; 5 Department of Medicine, College of Medicine, National Cheng Kung University, Tainan, Taiwan; 6 Department of Microbiology and Immunology, College of Medicine, National Cheng Kung University, No. 1, University Road, Tainan, 70101 Taiwan; 7 Center of Infectious Disease and Signaling Research, National Cheng Kung University, Tainan, Taiwan

**Keywords:** Zika virus, Seroprevalence, Neutralization tests, Plaque reduction neutralization tests, Flaviviruses

## Abstract

**Background:**

We recently conducted a serosurvey of newly arrived workers in Taiwan from four Southeast Asian countries which revealed that 1% of the migrant workers had laboratory-confirmed recent Zika virus (ZIKV) infection. Taiwan, where *Aedes* mosquitoes are prevalent, has a close relationship with Southeast Asian countries. Up to now, 21 imported cases of ZIKV infection have been reported in Taiwan, but there has been no confirmed indigenous case. The aim of this serosurvey was to assess whether there was unrecognized ZIKV infections in Taiwan.

**Methods:**

A total of 212 serum samples collected in a cross-sectional seroepidemiologic study conducted during the end of the 2015 dengue epidemic in Tainan, Taiwan, were analyzed. Anti-ZIKV IgM and IgG were tested using commercial enzyme-linked immunosorbent assays (ELISAs). Plaque reduction neutralization tests (PRNTs) for ZIKV and four dengue virus (DENV) serotypes were performed for samples with positive anti-ZIKV antibodies. A confirmed case of ZIKV infection was defined by ZIKV PRNT_90_ titer ratio ≥ 4 compared to four DENV serotypes.

**Results:**

The mean age of the 212 participants was 54.0 years (standard deviation 13.7 years), and female was predominant (67.0%). Anti-ZIKV IgM and IgG were detected in 0 (0%) and 9 (4.2%) of the 212 participants, respectively. For the 9 samples with anti-ZIKV IgG, only 1 sample had 4 times higher ZIKV PRNT_90_ titers compared to PRNT_90_ titers against four dengue virus serotypes; this individual denied having traveled abroad.

**Conclusions:**

The results suggest that undetected indigenous ZIKV transmission might have occurred in Taiwan. The findings also suggest that the threat of epidemic transmission of ZIKV in Taiwan does exist due to extremely low-level of herd immunity. Our study also indicates that serological tests for ZIKV-specific IgG remain a big challenge due to cross-reactivity, even in dengue non-endemic countries.

## Background

Zika virus (ZIKV), first isolated from a sentinel rhesus macaque in the Zika Forest in Uganda in 1947, is a flavivirus predominantly transmitted by *Aedes* mosquitoes [1, 2]. The majority of ZIKV infections are asymptomatic or present with mild, self-limited disease with symptoms of fever, maculopapular rash, arthralgia or nonpurulent conjunctivitis. As a result, ZIKV infections were seldom investigated in the past and might have been mistakenly attributed to dengue virus (DENV) due to clinical similarity and cross-reactivity in serologic testing [2, 3]. Being almost unnoticed for more than 60 years, ZIKV infection recently gained prominence due to several alarming epidemics in Pacific Islands and Latin America with potentially severe complications, including Guillain-Barré syndrome in adults and congenital anomalies in offspring of mothers who were infected during pregnancy [4]. As a result, the World Health Organization (WHO) declared the ZIKV epidemics a Public Health Emergency of International Concern in February 2016.

In Southeast Asia, serosurveys using neutralization assays in the 1950s provided evidence of ZIKV circulation in Malaysia, Philippine, Thailand, and Vietnam [5]. The first isolation of ZIKV was from *Aedes aegypti* in Malaysia in 1966 [6], and the first confirmed human case of ZIKV infection was documented in Indonesia in 1977 [3, 7]. However, the true disease incidence of ZIKV infections in Southeast Asia remains largely unknown because of the challenges of serological diagnosis due to cross-reactivity [3]. Virus-specific neutralization tests are more accurate to detect anti-ZIKV antibodies, but they are seldom being used in large epidemiologic studies because they are labor-intensive, time-consuming and expensive. Nevertheless, we recently conducted a serosurvey of 600 newly arrived workers from four Southeast Asian countries including Indonesia, Philippines, Thailand, and Vietnam in Taiwan using commercial enzyme-linked immunosorbent assays (ELISAs) and plaque reduction neutralization tests (PRNTs) for further confirmation [8]; the results showed that 6 (1%) of the migrant workers had laboratory-confirmed recent ZIKV infection defined using the World Health Organization criteria [8], suggesting the incidence of ZIKV infection in Southeast Asian countries may be severely underestimated and the risk of transmitting ZIKV from migrant workers and travelers from Southeast Asia cannot be neglected.

Taiwan, located in East Asia with the Tropic of Cancer lying across nearly centrally, has a population of over 23 million. Southern Taiwan belongs to the tropical climate zone where both *Aedes aegypti* and *Aedes albopictus* are prevalent, while northern and central Taiwan belongs to the sub-tropical climate zone where only *Aedes albopictus* can be found [9]. Currently, there has been no confirmed indigenous case of ZIKV infection in Taiwan, but 21 imported cases have been reported up to June 2019. With a very close relationship with Southeast Asian countries due to geographical proximity, Taiwan could face a potential risk of ZIKV outbreaks. Currently, more than 700,000 migrant workers from Southeast Asia live and work in Taiwan, and the number of visitors from Southeast Asia is over 2 million per year [10]. Although dengue is considered not to be endemic in Taiwan, dengue outbreaks of various sizes occur almost every year in southern Taiwan, presuming to be triggered by either unrecognized inapparent local infection [11] or by DENV-infected travelers coming from nearby Southeast Asian countries and subsequently disseminated through mosquitoes [12]. Similar to DENV, the frequent human migration and the presence of *Aedes* mosquitoes in Taiwan may also provide a suitable environment for autochthonous ZIKV transmission which may not be easily detected. The aim of this study was to investigate the seroprevalence of ZIKV-specific IgM and IgG to assess whether unrecognized ZIKV transmission had occurred in Taiwan.

## Methods

This study utilized a subgroup of blood samples collected in a cross-sectional seroepidemiologic study conducted during the end of the 2015 dengue epidemic caused by DENV serotype 2 (DENV2) in Tainan City, Taiwan [13]. The detailed methods for participant recruitment have been previously described [13]. In brief, a total of 1391 adult volunteers were recruited from three administrative districts with high dengue incidence and two districts with intermediate dengue incidence in Tainan. The study objectives and procedures were well explained to all the adult participants before obtaining written consent from them. Basic demographic information and history of DENV infection were obtained using a questionnaire. Questions used in the original survey are shown (Additional file 1: Appendix 1). Blood samples were collected and stored on ice during transport, processed, and then processed and stored at − 80 °C before serological testing. In the original serosurvey, anti-DENV IgM and IgG were tested using a commercial IgM capture ELISA (Standard Diagnostic, Kyonggi-do, South Korea) and an indirect IgG ELISA (Focus Diagnostics, Cypress, CA, USA) [13].

In this study, we selected samples collected from West Central District to test for anti-ZIKV IgM and IgG using commercial ELISA kits (Euroimmun, Luebeck, Germany) to investigate whether there was any undetected ZIKV transmission in Tainan during and before this dengue outbreak. This district was selected because it had the highest dengue incidence in the 2015 epidemic, and presumably might have higher vector density and other risk factors suitable for ZIKV transmission. There were 226 participants in this district in the original study, but 14 samples were without adequate residual volume. Therefore, only 212 samples were tested in this study. There was no significant difference in age, sex distribution, seroprevalence of anti-DENV IgM and IgG between those with and without adequate residual samples. All the tests were performed according to the manufacturer’s instruction [14]. The interpretation was based upon a ratio of the extinction value of a participant’s sample over the extinction value of a calibrator: positive (≥1.1), negative (< 0.8) and borderline (< 1.1 and ≥ 0.8) according to the instructions accompanied with the assays.

For samples with positive IgM or IgG against ZIKV, PRNTs for two ZIKV strains (strain MR766 and one clinical isolate from an imported case who was infected in Thailand in 2016) and all four DENV serotypes (DENV1 – 4; DENV1: strain Hawaii, DENV2: strain 16,681, DENV3: strain H87, DENV4: strain H241) were performed in parallel for further confirmation. The reason why two ZIKV strains were used in this study was that although most of imported cases of ZIKV infection in Taiwan were from Southeast Asia, imported cases from Latin America and the Caribbean as well as Africa were also reported. All viruses used for the PRNT assays were produced from Vero cells. Vero cells and baby hamster kidney fibroblasts (BHK-21) cells were prepared for ZIKV and DENV PRNT, respectively. Two-fold serial dilutions of sera beginning with a 1:40 dilution were utilized for standard PRNT assays according to previously published methods [15]. Sera from people negative for DENV and ZIKV IgG determined by ELISA were performed in parallel as negative controls. Titers required to reduce viral plaques by 50 and 90% compared with controls (PRNT_50_ and PRNT_90_) were determined by nonlinear regression curve fitting using four-parameter logistic-log (Sigmoidal 4PL) in GraphPad Prism version 7 for Windows (GraphPad Software, La Jolla, CA, USA). If the titer could not be calculated due to poor curve fitting, it was expressed as the last serum dilution showing a ≥ 50% and ≥ 90% reduction in plaque counts as compared with controls, respectively. A confirmed case of recent ZIKV infection was defined by positive anti-ZIKV IgM and ZIKV PRNT_90_ titer ratio ≥ 4 compared to four DENV serotypes as defined by the World Health Organization [16]. A confirmed case of past ZIKV infection was defined using the same PRNT titer ratio criteria but negative anti-ZIKV IgM [17].

## Results

The characteristics of the 212 study subjects were shown in Table 1. The mean age of the study population was 54.0 years (range 23 – 86 years, standard deviation [SD] 13.7 years), and female was predominant (67.0%). Twenty-six (12.3%) participants reported that they had been diagnosed with DENV infection before, and 19 (9.0%) people reported that the infection occurred in 2015. Seroprevalence of anti-DENV IgM and IgG was 23.1 and 42.5%, respectively.

**Table 1:** Characteristics of the study population

Characteristics	Number	Percent
Sex		
Male	70	33.0
Female	142	67.0
Age group, years		
20-34	24	11.3
35-49	47	22.2
50-64	94	44.3
≥ 65	47	22.2
Diagnosed with dengue in 2015		
Yes	19	9.0
No	193	91.0
Diagnosed with dengue in any time		
Yes	26	12.3
No	186	87.7
Anti-DENV IgM		
Yes	49	23.1
No	163	76.9
Anti-DENV IgG		
Yes	90	42.5
No	122	57.5

Anti-ZIKV IgM and IgG were tested using commercial ELISA kits. All 212 specimens showed negative for anti-ZIKV IgM. As a whole, 9 samples (4.2%) were positive for anti-ZIKV IgG, but five samples (2.4%) showed borderline results and were therefore presumed to be negative in this analysis. Seven out of the 9 samples positive for anti-ZIKV IgG were also positive for anti-DENV IgG and anti-DENV IgM, one sample was only positive for anti-DENV IgG, and the other one was negative for both anti-DENV IgM and IgG. As such, these 9 samples were subjected to PRNT to differentiate the identity of the infected virus. The PRNT and ELISA results of the 9 samples with positive anti-ZIKV IgG were shown in Table 2. Eight out of 9 samples did have PRNT_90_ titers to DENV, mainly DENV2, which was consistent with the 2015 DENV2 outbreak in southern Taiwan. Interestingly, although none of the samples had PRNT_90_ titer to the recent clinical Thailand isolate, 5 out of 9 samples had PRNT_90_ titers to ZIKV strain MR766 greater than 40. By definition, only 1 sample (0.5%) had 4 times higher ZIKV PRNT_90_ titers compared to PRNT_90_ titers against DENV1 – 4, suggesting that this individual had been previously infected by ZIKV. The one sample positive for anti-ZIKV IgG, but negative for both anti-DENV IgM and IgG did not have any PRNT titers to either DENV or ZIKV.

**Table 2:** Serological test results of the 9 participants with positive anti-ZIKV IgG and negative anti-ZIKV IgM

			ELISA		PRNT_50_						PRNT_90_					
No	Age- ranges, years	Sex	DENV IgM	DENV IgG	DENV1	DENV2	DENV3	DENV4	MR766	Thailand strain	DENV1	DENV2	DENV3	DENV4	MR766	Thailand strain
1	70-74	M	+	+	< 40	637	< 40	199	> 2560	< 40	< 40	479	< 40	< 40	401	< 40
2	20-24	F	+	+	1335	1139	120	285	239	< 40	288	436	< 40	< 40	< 40	< 40
3	75-79	F	+	+	1445	> 2560	161	< 40	< 40	< 40	< 160	> 2560	< 40	< 40	88	< 40
4	75-79	M	+	+	< 40	1418	1127	< 40	< 40	< 40	< 40	320	320	< 40	< 40	< 40
5	55-59	F	+	+	886	1353	230	< 40	< 40	< 40	433	320	< 40	< 160	< 40	< 40
6	70-74	M	+	+	305	> 640^a^	116	< 40	> 640^a^	160	160	157	< 40	< 40	207	< 40
7	75-79	F	+	+	< 40	118	< 40	< 40	> 640^a^	< 40	< 40	44	< 40	< 40	160	< 40
8	55-59	M	–	+	202	198	< 40	< 40	>640^a^	188	40	52	< 40	< 40	>640^a^	< 40
9	70-74	M	–	–	< 40	< 40	< 40	< 40	< 40	< 40	< 40	< 40	< 40	< 40	< 40	< 40

*ELISA* enzyme-linked immunosorbent assay, *PRNT* plaque reduction neutralization test, *DENV* dengue virus, *DENV1* dengue virus serotype 1, strain Hawaii, *DENV2* dengue virus serotype 2, strain 16,681; *DENV3* dengue virus serotype 3, strain H87, *DENV4* dengue virus serotype 4, strain H241, *MR766* Zika virus strain MR766. Thailand strain, one Zika virus isolate from an imported subject who got infection in Thailand

^a^Additional dilutions were not performed due to inadequate amount of residual samples

## Discussion

In this study, we investigated the seroprevalence of IgM and IgG against ZIKV using commercial ELISA kits among 212 adult individuals recruited during the end period of the 2015 dengue epidemic in Tainan, Taiwan. All samples were negative for anti-ZIKV IgM, including those 49 samples positive for anti-DENV IgM, suggesting that there was no undetected recent or ongoing ZIKV transmission during 2015 dengue epidemic in Tainan. The results also suggest that the commercial anti-ZIKV IgM ELISA is very specific with minimum cross-reactivity to DENV infection in dengue non-endemic countries. As for anti-ZIKV IgG, 9 samples were positive, among which, seven also had positive anti-DENV IgM and high PRNT_90_ titers against DENV2; these subjects were likely infected during the 2015 DENV2 epidemic in Tainan. In addition, most of them also had neutralizing antibodies against other DENV serotypes. As a result, the positive anti-ZIKV IgG among these subjects was more likely to be due to cross-reactivity to multiple DENV infections [18]. These findings also suggest that commercial ELISA kits for anti-ZIKV IgM can be a good diagnostic test for ZIKV infection in Taiwan and probably in other dengue non-endemic countries since cross-reactivity is of less concern; however, serological tests for ZIKV-specific IgG still remain a challenge, even in dengue non-endemic countries.

Only one sample fulfilled the criteria of ZIKV PRNT_90_ titer ratio ≥ 4-fold higher than DENV1 – 4 titers, suggesting this participant might have been previously infected by ZIKV, though the possibility of a false positive result could not be completely ruled out since high PRNT_50_ titers against DENV1 and DENV2 were also observed. However, one recent longitudinal study revealed that in subjects with ZIKV infection, the highest neutralizing antibody titers were to ZIKV, with low-level cross-reactivity to DENV1 – 4 even in people with previous DENV infection, indicating that neutralizing antibody titers can be used to differentiate between ZIKV and DENV infections correctly when all viruses are analyzed simultaneously [19]. As a result, this participant had strong serological evidence of past ZIKV infection. This participant was a 57-year-old male, and he denied having traveled abroad or history of dengue, Japanese encephalitis, infection by other flaviviruses, and autoimmune diseases on a follow-up call. Therefore, this case suggested that unrecognized indigenous ZIKV transmission might have occurred in Taiwan, though the possibility of a false positive result could not be completely excluded. Future serosurveys to include more people in more areas with random sampling are required to confirm to the finding. Alternatively, samples from patients with acute fever and rash but unknown etiology should be tested for ZIKV infection to further investigate whether local transmission of ZIKV has occurred in Taiwan.

PRNT is an effective method to differentiate infections caused by different flaviviruses. However, one major concern on the interpretation of our data was that the PRNT_90_ titers against the two used ZIKV strains were very different. One systematic review shows that PRNT titers against different strains within a single DENV serotype can vary substantially [20], suggesting that this scenario could also be true for ZIKV. MR766 is an African-lineage laboratory-adapted ZIKV strain, while the Thailand strain was a 2016 clinical isolate from an imported case infected in Thailand, which was kindly provided by Taiwan Centers for Disease Control. These two strains are very different and thus the PRNT titers may differ. In addition, we observed that the African ZIKV strain (MR766) was more reactive in the PRNT and provided overall higher titers compared to the 2016 clinical Thailand isolate, while only a couple of specimens had PRNT_50_ titers against the Thailand isolate. Interestingly, higher PRNT titers against the MR766 strain than against the 2016 Thailand isolate were also observed in most of the migrant workers from Southeast Asian countries in our previous study [8], which, to our surprised, was an unexpected. A recent report in the rhesus macaque model shows that a single mutation in antibody recognition epitope domain of Zika viral E protein can result in ineffective neutralization by human monoclonal antibody [21]. Comparison of the antibody recognition epitope sequences of both MR766 and the clinical Thailand strains retrieved from the National Center for Biotechnology Information (NCBI) website (https://www.ncbi.nlm.nih.gov), the position at 393 of E protein is different, in which the amino acid is E in MR766, while D in Thailand strain. The positional difference is in line with the report in Rhesus Macaque study [21], which may provide an explanation on the low PRNT titers against the 2016 clinical Thailand strain in this study and in migrant workers from Southeast Asia [8].

One sample positive for anti-ZIKV IgG but negative for both anti-DENV IgM and IgG did not have any PRNT titers against either ZIKV or DENV. Although the reasons remain to be investigated, a few scenarios could be accounted for the odd phenomenon; for example, false positive due to the individual has autoimmune diseases [22] or infection by other flaviviruses. Nonetheless, the result also suggests that development of better and precision diagnostic tools for ZIKV infections are urgently needed.

Seroprevalence studies are an important tool to assess the disease burden, epidemiology of flavivirus infections and herd immunity. Recently, a number of serosurveys of ZIKV infections have been conducted in Oceania, Africa, Latin America, and the Caribbean. The seroprevalence was high in Micronesia (73%) [23], Brazil (63.3%) [24], Nicaragua (36 – 56%) [25], French Polynesia (49%) [26], Martinique (42.2%) [27], Bolivia (0 – 39%) [28], Suriname (35.1%) [29], French Guiana (18.8%) [30], Saudi Arabia (12.68%) [31], and Nigeria (10%) [32], but was less than 10% in Laos (9.9%) [33], Indonesia (9.1%) [17], Zambia (6.1%) [34], Cameroon (5%) [35], Rwanda (1.4%) [36], and Kenya (0.24 – 7.11%) [37]. However, the laboratory assays used were varied across the studies. Some studies only used ELISA to identify anti-ZIKV antibodies without performing neutralization assays for further confirmation, and thus the false-positive rate could be high due to cross-reactivity, especially in countries with endemic circulation of other flaviviruses. Although the other studies detected ZIKV neutralizing antibodies, different laboratory methods and criteria were adopted to define ZIKV seropositive. In addition, the population selected and age distribution in these serosurveys also differed significantly. Therefore, seroprevalences from different studies may not be directly comparable [38]. In our study, 4.2% of the participants were anti-ZIKV IgG positive using commercial ELISA kits, but only 0.5% were considered true ZIKV seropositive using stringent PRNT_90_ criteria. To be noted, we selected people from the district with the highest dengue incidence in this study because they should also have a higher chance of ZIKV infection since DENV and ZIKV share similar transmission routes. Therefore, seroprevalence of ZIKV infection should be even lower in other parts of Taiwan. Although the results suggest that seroprevalence in Taiwan is low compared to other countries, this study provides serological evidence of unrecognized indigenous ZIKV transmission in Taiwan. In addition, our findings also indicate that ZIKV outbreaks may occur in Taiwan due to the extremely low-level of herd immunity.

There were several limitations to this study. The sample size of the study was small and the study participants were not randomly selected; therefore, the study population might not be representative of the general population in Taiwan. In addition, serological tests including ELISA and PRNT were not performed for other related flaviviruses, such as Japanese encephalitis virus and yellow fever virus. However, the incidence of infection by other flaviviruses was extremely low in Taiwan. Therefore, performing additional serologic testing for other flaviviruses would be time-consuming but might not add much value to this study.

## Conclusion

In conclusion, this study is the first serosurvey of ZIKV infection in Taiwan. Results showed 9 (4.2%) of 212 participants had positive anti-ZIKV IgG using commercial ELISAs but only 1 (0.4%) participant was considered to be true past ZIKV infection defined by PRNT_90_. This participant denied having traveled abroad, suggesting that unrecognized indigenous ZIKV transmission might have occurred silently in Taiwan. The results also suggest that the threat of epidemic transmission of ZIKV in Taiwan does exist due to extremely low-level herd immunity in general population due to the prevalence of *Aedes* mosquitoes, and frequent human travels from and to Southeast Asia. Our study also indicates that commercial ELISA kits for anti-ZIKV IgM can be a good diagnostic test for acute ZIKV infection in dengue non-endemic countries; however, serological tests for ZIKV-specific IgG still remain a big challenge, even in dengue non-endemic countries.

## Supplementary information

**Additional file 1.** Questions used in the original survey.

## Data Availability

The datasets used and/or analyzed during the current study are available from the corresponding author on reasonable request.

## Abbreviations

DENV: Dengue virus
ELISA: Enzyme-linked immunosorbent assay
PRNT: Plaque reduction neutralization test
ZIKV: Zika virus
