# Supplementary material for: Low seroprevalence of Zika virus infection among adults in Southern Taiwan
Source: BMC Infect Dis. 2019 Oct 23;19:884. doi: 10.1186/s12879-019-4491-4 (PMC6813068; doi:10.1186/s12879-019-4491-4)
Supplement: Supplementary file 1 — Additional file 1. Questions used in the original survey. [file 12879_2019_4491_MOESM1_ESM.docx]

**Low seroprevalence of Zika virus infection among adults in southern Taiwan**

**Appendix 1**

*Questions used in the original serosurvey*

1. Gender: □ Male □ Female
2. Year of birth:
3. Address:
4. Have you ever been diagnosed with dengue virus infection?

□ No

□ Yes. Please fill in the time and place of each infection.

a. First time of infection: Year Month ; place of infection:

b. Second time of infection: Year Month ; place of infection:

c. Third time of infection: Year Month ; place of infection:

1. Have your family members living with you ever been diagnosed with dengue virus infection?

□ No

□ Yes. Please fill in the time and place of each infection.

a. First time of infection: Year Month ; place of infection:

b. Second time of infection: Year Month ; place of infection:

c. Third time of infection: Year Month ; place of infection:
